# The Impact of Cytokines on Neutrophils’ Phagocytosis and NET Formation during Sepsis—A Review

**DOI:** 10.3390/ijms23095076

**Published:** 2022-05-03

**Authors:** Barbara Gierlikowska, Albert Stachura, Wojciech Gierlikowski, Urszula Demkow

**Affiliations:** 1 Department of Laboratory Diagnostics and Clinical Immunology of Developmental Age, Medical University of Warsaw, Zwirki i Wigury 63a, 02-097 Warsaw, Poland; urszula.demkow@wum.edu.pl; 2 Centre for Preclinical Research, Department of Methodology, Medical University of Warsaw, Banacha 1b, 02-097 Warsaw, Poland; albert.stachura@wum.edu.pl; 3 Doctoral School, Medical University of Warsaw, Zwirki i Wigury 61, 02-091 Warsaw, Poland; 4 Department of Internal Medicine and Endocrinology, Medical University of Warsaw, Banacha 1a, 02-097 Warsaw, Poland; wojciech.gierlikowski@wum.edu.pl

**Keywords:** neutrophil, sepsis, cytokine, phagocytosis, NET formation

## Abstract

Sepsis is an overwhelming inflammatory response to infection, resulting in multiple-organ injury. Neutrophils are crucial immune cells involved in innate response to pathogens and their migration and effector functions, such as phagocytosis and neutrophil extracellular trap (NET) formation, are dependent on cytokine presence and their concentration. In the course of sepsis, recruitment and migration of neutrophils to infectious foci gradually becomes impaired, thus leading to loss of a crucial arm of the innate immune response to infection. Our review briefly describes the sepsis course, the importance of neutrophils during sepsis, and explains dependence between cytokines and their activation. Moreover, we, for the first time, summarize the impact of cytokines on phagocytosis and NET formation. We highlight and discuss the importance of cytokines in modulation of both processes and emphasize the direction of further investigations.

## 1. Sepsis

Sepsis is a life-threatening organ dysfunction caused by a dysregulated host response to infection, as a consensus definition states [1]. The first consensus, from 1991, defined sepsis as a systemic inflammatory response (SIRS) to infection [2]. The term “SIRS” is currently not considered an accurate descriptor of sepsis pathobiology [1], as sepsis is now linked not only with pro-inflammatory, but also with anti-inflammatory response [3].

Clinical symptoms, laboratory results, and radiology results are nonspecific; microbiological cultures need time and are negative in most of the cases, thus a constellation of findings is often needed to make the diagnosis [4]. Even efforts to support clinicians with automatic tools to improve diagnosis led to rather disenchanting results [5]. Sepsis may manifest as septic shock, both conditions being considerable worldwide healthcare problems, leading to death in between 15 to over 30% of affected individuals [6,7]. Evidence-based guidelines for sepsis management are frequently updated by experts leading the initiative Surviving Sepsis Campaign, with its currently up-to-date version from 2021 [4]. However, sepsis is not a specific illness but rather a syndrome encompassing a still-uncertain pathobiology [1]. Additionally, a heterogeneity of patients’ groups impedes studies on sepsis [8]. Authors of the consensus definition of sepsis aimed for greater consistency of epidemiologic studies and clinical trials, and to facilitate earlier recognition and rapid management of patients with sepsis [1]. As early diagnosis and treatment improve the outcomes in patients, it is of paramount importance to better understand the underlying mechanisms orchestrating sepsis.

Virtually all infectious agents may cause sepsis. Importantly, microbiological identification is possible in less than half of the cases [9]. These facts contribute to the fact that the range of symptomatology and clinical manifestation vary considerably. Sepsis may originate from community locations or be a consequence of hospital stay, especially at the intensive care units, where the risk of infection increases over time [10]. Sites of infection that are the most common origin of sepsis are the lung (64%), abdomen (20%), bloodstream (15%), as well as renal and genitourinary tracts (14%) [11].

Pathophysiology of sepsis stems from dysregulated immunological host response to the invading pathogen. One pathway responsible for this phenomenon is linked to pattern-recognition receptors, including Toll-like receptors, which activate in response to microbial pathogen-associated molecular patterns [12]. This prompts the release of both pro- and anti-inflammatory cytokines mediated by translocation and activation of nuclear factor κB (NF-κB) [13]. Complement and clotting cascades are also activated, sometimes leading to generation of microthrombi [14]. The clinical image varies from massive thromboembolism to fibrin deposition in the vascular bed. A significant complication of sepsis is disseminated intravascular coagulation (DIC), which manifests with microthrombosis and simultaneous bleedings [15].

In the past, sepsis was considered as an overwhelming systemic proinflammatory response to infection with subsequent deterioration of immunological function, characterized by anergy, lymphopenia, and subsequent infections [14]. Indeed, higher incidence of nosocomial infections is observed in patients who survive the initial phase of sepsis—these include fungal infections or reactivation of latent viruses [16]. Such a state is at least partly associated with deficient T-cell response, mediated by activity of the programmed cell death protein 1 and interleukin 7 (IL-7) [3]. Newer data suggest that the proinflammatory and immunosuppressive factors act synergistically during sepsis and their dynamics depend on various circumstances. Hosts may have genetical proclivity for driving this balance either way or may simply have underlying comorbidities, compromising the functionality of immunological system. The pathogens may also affect the host differently, depending on the type, virulence, and burden [16].

Microcirculatory dysfunctions developing during sepsis have been associated with poor prognosis in patients [17]. Interference with systemic blood flow to organs—mainly by the means of vasodilation—frequently occurs in sepsis, being a core dysfunction during septic shock development. Nitric oxide is believed to play a key role in this phenomenon [18]. The precise mechanisms of organ damage and cellular dysfunction in sepsis are, however, not fully understood and are subject of scientific investigations [19].

Finally, sepsis is frequently accompanied by severe ischemia, originating from a dysregulation between deteriorated oxygen distribution and tissue demand. Clinically it manifests as a low blood pressure (i.e., mean arterial pressure <65 mm Hg) and increased serum lactate level despite absence of hypovolemia [1]. On the cellular level, oxygen has diminished availability despite sufficient delivery—a phenomenon called cytopathic hypoxia, caused by mitochondrial dysfunction, and leading to decreased adenosine triphosphate synthesis [20]. These mechanisms, together with above-mentioned microcirculatory abnormalities and apoptosis are believed to contribute to multiple organ failure in patients with sepsis [21].

## 2. The Role of Neutrophils during Sepsis

Neutrophils play some key roles in defense against pathogens, especially bacteria. In sepsis, however, some of the mechanisms do not work properly, which may lead to impaired immunological response or tissue damage. Neutrophils (or polymorphonuclear cells—PMNs) derive from bone marrow and—when matured—are released to the bloodstream. Their life span is relatively short, and they have no proliferative capabilities [22]. PMNs exert their function by several means. The primary killing mechanisms are phagocytosis, degranulation, and release of neutrophil extracellular traps (NETs).

When neutrophils encounter pathogens, they encapsulate them in phagosomes and kill them, using NADPH oxidase-dependent mechanisms (primarily reactive oxygen species, ROS) or antibacterial proteins [23]. These proteins are released either directly into the phagosome or into the surroundings, acting on both intra- and extracellularly localized microbes. Another way to dispose of pathogens is releasing NETs, which consist of core DNA, to which histones, proteins, and enzymes (such as myeloperoxidase—MPO—and elastase) are attached. Such traps immobilize pathogens and facilitate phagocytosis. Above-mentioned attached molecules may also directly damage microbes, which they have contact with [24,25]. Under normal circumstances, cytokines, such as TNF-α, IL-1, IL-2, IL-6, and IL-8 trigger the neutrophil-endothelial cell adhesion, facilitating cells’ migration to the inflamed site [14].

Neutrophils control the course of infections by (1) migrating to inflamed site and (2) proper execution of the killing mechanisms, aimed at pathogens. In sepsis, however, both these components are impaired and lead to either insufficient response or to collateral damage inflicted to surrounding tissues [26]. The degree to which neutrophils’ phagocytosis and NETosis are impaired during sepsis will be described in more detail in separate chapters together with the underlying mechanisms.

Before neutrophils begin eliminating pathogens, they must first reach the target site. Chemokines are a family of cytokines, which facilitate the process of recruiting neutrophils to the inflamed area [27]. In vivo experiments in mice showed that during sepsis PMNs are not properly recruited to the target site despite a substantial amount of produced chemokines [28]. Failure to curb the local inflammation leads to a systemic infection.

One of the mechanisms underlying impaired recruitment is the internalization of CXCR2 (CXC receptor 2) in circulating neutrophils [29]. Chemokine receptors belong to G protein-coupled receptors (GPCRs), which become inactive in response to phosphorylation by GPCR kinases (GRKs). These, in turn, are upregulated when a big number of pathogen-derived ligands bind with Toll-like receptors (TLR2, TLR4, and TLR9). Therefore, activated GRKs phosphorylate a domain of CXCR2, internalizing it [30]. Importantly, in the face of pro-inflammatory response, adequate activation of TLRs does not down-regulate CXCR2 and neutrophils may be appropriately recruited to the inflamed site, establishing the local immune response [31].

Other factors influencing CXCR2/CXCL2 (CXC ligand 2) dynamics are TNF-α and NO [32,33]. Neutrophils treated with the former show lowered chemotaxis and migration capability toward CXCL2. Both TLR- and TNF-dependent pathways upregulate inducible NO synthase, which could potentially lead to activating GRK2, with subsequent phosphorylation of CXCR2 and its internalization. Moreover, NO reduces the levels of adhesion molecules on both endothelial cells and neutrophils [28]. There are other mediators at play when it comes to neutrophil migration during sepsis, such as lectin-like oxidized low-density lipoprotein receptor (LOX)-1, peroxynitrite, and the acute-phase alpha-1 acid protein, all of which impair neutrophils’ ability to reach the target site [34,35,36]. On the contrary, IL-17 seems crucial to recruiting neutrophils to the site of infection during sepsis [37].

Apart from significant migration dysregulation, neutrophils exhibit some deleterious functions during sepsis [26]. Firstly, activated neutrophils resident in the capillary beds cluster and occlude the lumen, which may lead to ischemia [38]. Moreover, they may migrate to vital organs, where they release pro-inflammatory and lytic factors, causing damage to local tissues [39]. Interestingly, neutrophils may migrate to lungs when they receive signals mediated by previously discussed CXCR2 receptor [40]. Such a potential contradiction may be explained by differences in the severity of infection. Another receptor is also involved in recruiting neutrophils to vital organs, such as heart or kidneys—CCR2 [41,42]. Signals transmitted through TLR2 and TLR4 during sepsis induce the expression of CCR2 on the neutrophils’ surface. CCR2 levels are correlated with the disease severity, and neutrophils isolated from non-surviving patients show greater expression of CCR2 than these from surviving patients [43].

As mentioned previously, NETosis is an important bacteria-killing mechanism. It seems to be less efficient in sepsis, however, as bacterial loads noted in mice, which had impaired NET formation capacity, were similar to those in control animals [44]. Importantly, excessive NET formation may in fact lead to a state of increased coagulation, as NETs activate the vascular endothelium and interact with activated platelets [45,46]. Some of the molecules attached to DNA strands, such as MPO or histones, may inflict damage to epithelium. Histones themselves may prompt TLR2- and TLR4-dependent signals via MyD88 pathway, leading to increased pro-inflammatory cytokine production [47,48]. All these effects seem to act destructively on the surrounding tissues and organs, contributing to the development of multiple organ failure.

Hypoperfusion and tissue hypoxia were discussed as crucial factors deteriorating the prognosis in patients with sepsis. Microcirculation occluded with neutrophils is associated with these phenomena [49]. Apart from direct unfavorable effect exerted on epithelium, neutrophils also induce expression of NO, lowering blood pressure [50]. Their products also contribute to generating peroxynitrite, an oxidant agent, which causes substantial changes to structure and function of molecules, related to myocardial dysfunction during sepsis [51].

## 3. Cytokines—Overview

Cytokines are molecules that regulate various processes, such as proliferation, differentiation, and cell mobility. By affecting a broad spectrum of cells, cytokines mediate inflammatory and immune reactions and participate in the regulation of hemopoiesis. Over one hundred and several dozen cytokines have already been identified. Their number is constantly increasing. There is no single cytokine classification scheme. Due to structural similarities, there are: type 1 cytokines (hematopoiesis), type 2 cytokines (interferons and IL-10 families), chemokines, and the TNF-α superfamily [52,53].

Some cytokines are often referred to as interleukins to reflect their effects between different leukocyte populations. With time, however, it turned out that many interleukins can be secreted by other types of cells (keratinocytes, fibroblasts) and that they can influence not only leukocytes, but almost any other type of cell. Although the cytokines initially secreted by monocytes were referred to as monokines and those that produced lymphocytes were called lymphokines, these terms are not currently used as none of the cytokines are secreted exclusively by a single cell type [52,54].

The characteristic features of cytokines are (1) pleiotropy, i.e., the ability of a given cytokine to affect many different cells and induce different effects, and (2) redundancy, i.e., the ability of different cytokines to cause the same effect. Some cytokines can antagonize each other, blocking the biological effects of each other. Other cytokines acting simultaneously on the same cells achieve a synergistic effect. Another property of cytokines is the ability to induce positive and negative feedbacks [52,53].

When analyzing the participation of cytokines in the activation, proliferation, and differentiation of cells, it should be remembered that these processes are also regulated by direct intercellular interactions [55,56]. Some cytokines are produced initially as membrane molecules involved in the direct activation of target cells. An example of this type of interaction is TNFs, which initially occurs in the form of a membrane protein but is subsequently secreted into the environment by a suitable metalloproteinase [57]. Cytokines can act on the same cells that secrete them (autocrine effect), cells in the immediate vicinity (paracrine effect), or on cells in other organs (endocrine effect) [58].

It may seem that due to such a large number of cytokines and their intricate interactions, the immune response should be a chaotic rather than an organized phenomenon. However, the sensitivity of cells to cytokines depends on the prior recognition of the antigen. Thus, when produced at high concentrations, cytokines will not act on all lymphocytes, but only on those cells that specifically recognize the antigen and are ready to perform effector functions. Moreover, the action of many cytokines is related to their local secretion, i.e., to the immune synapse, thanks to which they achieve local high and effective concentration.

### 3.1. Receptors for Cytokines

Cytokines can exert their functions only due to the presence of receptors on target cells. Research into the structure of these receptors and their signaling pathways has answered many questions about the role of cytokines in the functioning of the immune system and the other systems they act on [59].

A characteristic feature of most known cytokine receptors is that their extracellular fragments contain characteristic domains. These domains are responsible for the ligand binding specificity, but also influence the way the signal will be transduced after cytokine binding. The second characteristic feature of cytokine receptors is the presence of intracellular domains, which are directly responsible for initiating signals in the cell. The extracellular part is connected to the cytoplasmic section by transmembrane fragments of the receptor. Despite the distinct differences in the structure of cytokine receptors, these receptors can be divided into five different types: Ig-like receptors, class 1 cytokine receptors (hematopoietin receptors), class 2 cytokine receptors (receptors for interferons and the IL-10 family), receptors for the TNF superfamily, and G-protein coupled receptors (chemokine receptors) [52,60].

Receptors that cross the cell membrane only once must be di- or trimerized-processes necessary for signaling. In this case, the cytokine acts as a molecule that creates a binder that allows the horizontal shift of the receptor subunits in the plane of the cell membrane. Only then, as the cytoplasmic sections of the receptors come closer to each other, signal transmission is initiated due to the cross phosphorylation of these sections or proteins associated with them.

The binding of cytokines to receptors on the cell membrane leads to the activation of signal transduction pathways in the cell. The pathways of GTPases and MAP kinases, tyrosine kinases from the Src- and Tec-like families, and phosphatidylinositol-3-kinases (PI-3K) are involved here [61]. However, most cytokines activate the signal transduction pathway by JAK (Janus kinases) tyrosine kinases and STAT (signal transducers and activators of transcription) proteins. The latter, after activation, form dimers and translocate to the nucleus acting as transcription factors [62].

### 3.2. Sepsis and Cytokines

Current theories suggest that the sepsis may be associated with an early overwhelming innate immune response, characterized by dysregulation of protein mediators: activation and release of pro-inflammatory cytokines (TNF-α, IL-1β, IL-6, IL-8), and their receptors (IL-1RA, TNF-R1/2) and dysregulation of crucial molecules, which modulate immune response (e.g., MCP-1, HMGB-1, PD-1, CTLA-4, NGAL, MMP-9, TIMP2, PAI-1). Importantly, the recent studies provide convincing data that mitochondrial DNA (mtDNA) can influence the immune system through toll-like receptor 9 and inflammasomes. Clinical trials provide evidence that mtDNA is elevated in critically ill patients and is associated with mortality [63].

Recently drugs targeting cytokines signaling are extensively studied in COVID-19, as in acute respiratory distress syndrome (ARDS) phase symptoms in large part results from cytokine storm and collateral organ damage. Corticosteroids were some of the first investigated drugs and indeed, RECOVERY trial show favorable influence of dexamethasone [64]. According to National Institutes of Health guidelines (last update on 24 February 2022), IL-6 or Janus Kinase inhibitors (i.e., tocilizumab, sarilumab, or baricitinib, tofacitinib, respectively) may be added as a second immunomodulating drug. Evidence supporting use of tocilizumab mainly comes from REMAP-CAP trial [65]; nevertheless, other studies also favor the drug, as reviewed in [66]. Evidence speaking for baricitinib and tofacitinib are based on randomized controlled trials (COV-BARRIER [67], and STOP-COVID [68], respectively). Anakinra, IL-1β inhibitor, also seems to reduce mortality in the late phase of COVID-19, as reviewed in [66].

The most important sepsis mediators are presented at Table 1. Importantly, most of them are detectable in the blood’s serum.

The presence of cytokines is normally restricted to an area of injury. However, when a local infection spreads, a strong systemic reaction occurs, and signs of sepsis are apparent. Under such circumstances, mediators can be detected systemically, and may lead to septic shock. On the other hand, innate deficiency of cytokine release during acute severe infections leads to a rapid multiplication of the invading microorganisms, which results in reactions of the host consisting of pro-inflammatory and anti-inflammatory reactions (term SIRS is still used in this context, and term compensatory anti-inflammatory response syndrome (CARS) is used, respectively), which could ultimately lead to shock and death. An inadequate systemic inflammatory response is partially counterbalanced by sustained expression of potent anti-inflammatory mediator IL-10 [101,102].

Cytokines are small (8–26 kD), highly active molecules, which are synthesized primarily by the cells of the immune system. Concentrations of circulating pro-inflammatory cytokines are low or undetectable in healthy individuals but their production is stimulated during host invasion by pathogenic microorganisms. Four cytokines, TNF-α, IL-1β, IL-6, and IL-8 have been most strongly associated with sepsis. In human and experimental animal models of sepsis, cytokines are released in a sequential manner resulting in a “cytokine cascade” [103]. It is initiated when a stimulus, such as a Gram-negative bacterial endotoxin (e.g., lipopolysaccharides released by E. coli), induces production of the “early inflammatory cytokines”, such as TNF-α and IL-1β. TNF-α is regarded as a central mediator of immune regulation and of the pathophysiological changes associated with bacteremia and sepsis syndrome [101]. Plasma TNF-α concentrations are increased in patients with both Gram-negative and Gram-positive infectious diseases [102]. Overproduction of TNF-α correlates with enhanced properties of phagocytes. In contrast, IL-1β serum levels are only slightly increased during sepsis. The release of “early inflammatory cytokines” intensifies the production of the “late inflammatory cytokines”—IL-6 and IL-8. The mortality rate is significantly higher in patients who present with a high IL-6 serum level (above 1000 pg/mL). IL-6 concentration is recognized as a marker of sepsis with high specificity [104]. Redl and colleagues [105] showed that treatment with anti-TNF-α antibodies results in significantly decreased IL-8 concentrations in the bloodstream. The increased plasma IL-8 concentration in adult sepsis-occurring patients may correlate with mortality [106], however, not enough observations clearly confirm this hypothesis.

Proinflammatory cytokines play a crucial role in the activation of the host defense. However, various experimental studies have shown that an overwhelming production of these mediators can lead to vasodilation, increased vascular permeability, hypotension, multiple organ failure, dysregulation of other protein mediators and ultimately shock and death [107].

Although various pro-inflammatory cytokines contribute to the inflammatory cascade, other cytokines also display anti-inflammatory properties, serving to counterbalance a potentially inadequate proinflammatory state. IL-10 in particular has been implicated as the primary endogenous modulator of inflammatory response during sepsis. The importance of IL-10 production during sepsis has been well established in various sepsis models [108,109]. Gerard and colleagues [110] showed that treatment of mice with IL-10 before endotoxin administration could prevent endotoxin-induced mortality and diminish plasma TNF-α release. On the other hand, inhibition of IL-10 during course of sepsis may also be beneficial [111]. This may seem like a contradiction, resulting for example from inconsistent experimental models, but different roles of a single cytokine may result from interactions with other cytokines at different stages of the disease. Future studies are needed, as indicated by Mazer et al. [112].

The dysregulation of “cytokine balance” results in modulation of immune cells functions. The question is: how does the cytokine profile affect neutrophils? The role of neutrophils in sepsis and influence of selected cytokines affecting effector functions of neutrophils are discussed below.

## 4. The Impact of Cytokines on Phagocytosis Performed by Neutrophils

The most important cytokines classified as mediators of phagocytosis performed by neutrophils are summarized at Table 2.

Cytokine-like 1 (CYTL1) increased phagocytosis of activated neutrophils both in in vivo and in vitro models [113]. Authors hypothesized that CYTL1-enhanced phagocytosis of *Escherichia coli* by activated neutrophils is dependent on phosphorylation of protein kinase B (Akt). Additionally, CYTL1 also increased the release of ROS in LPS-stimulated neutrophils. ROS are powerful antimicrobial agents produced in phagosomes and phagolysosomes; thus, their concentration directly affects the efficiency of the whole phagocytosis process.

Onogawa et al. tested whether IL-6 affects phagocytosis efficiency during sepsis. Using mice model infected with *Staphylococcus aureus*, they proved that the augmentation of the IL-6 signal by recombinant IL-6 receptors (rIL6R) allows the functional recovery of phagocytes in a peritonitis murine model, and consequently improves their phagocytic functions. The authors noticed an increased uptake of *S. aureus* and phagosomal acidification, which favors bacteria killing and phagolysosomes’ formation.

The direct effect of IL-6 on phagocytosis and ROS production was also evaluated in vitro on neutrophils isolated from healthy volunteers [115]. IL-6 treatment resulted in a significantly increased bacterial uptake, as well as stimulation of ROS generation. Interestingly, co-treatment with IL-6 and TNF-α intensified ROS generation, but did not affect phagocytosis [115]. These findings underline that not just a single cytokine’s concentration matters, but rather concentrations of numerous cytokines, which together constitute cytokines’ profile, exert certain functions. Gaber et al. showed that inhibition of IL-6 signaling by tocilizumab affects phagocytosis in an oxygen-dependent manner—in normoxia tocilizumab stimulates, whereas in hypoxia it impairs phagocytosis [120]. Considering that in sepsis and especially in septic shock oxygen supply is reduced, rather the former result may be expected.

IL-10 was classified by Mittal et al. as a cytokine, which stimulates *E. coli* clearance [116]. Authors observed that administration of IL-10 during a high-grade bacteremia clears antibiotic-sensitive and -resistant *E. coli* from blood of infected mice. The suggested underlying mechanism was an increased expression of CR3 in phagocytes, which was caused by suppression of prostaglandin E-2 release. It may suggest that IL-10 mediates *E. coli* phagocytosis by precisely guiding bacteria via complement-dependent pathway.

Moreno et al. tested the role of IL-12 and IL-18 on neutrophil phagocytic functions in sepsis induced by cecal ligation and puncture (CLP) in a murine model [117]. Wild-type mice, as well as IL-18(−/−) mice were resistant to sepsis. On the contrary, IL-12(−/−) mice were susceptible to SL-CLP sepsis with high bacteria concentration, similarly to IFN-γ (−/−) mice. However, stimulating IL-12-deficient neutrophils with IFN-γ restored their phagocytic functions, stimulated NO production and more effective clearance of pathogens.

Increased level of IL-17 stimulates pathogen clearance but does not have a major impact on the inflammation pathology [118]. Another study was performed by van de Veerdonk et al. in two fungal-induced septic models. Intravenous infection with live *Candida albicans* and zymosan injection showed a protective role of IL-10 during *C. albicans* clearance. Although IL-10 did not protect against zymosan-induced organ failure, the role of IL-10 was classified as important but not crucial.

The next cytokine that affects phagocytosis during sepsis is IL-34 [119]. It was tested in two models: wild-type C57BL/6 mice were used for in vivo studies, and septic human patients and healthy volunteers were recruited to obtain blood for in vitro studies. IL-34 concentration was significantly elevated in human sepsis and puncture-induced experimental sepsis. Additionally, administration of IL-34 successfully increased chemotaxis of neutrophils and strengthened their phagocytic functions. Decreased IL-34 concentration increased mortality in mice model and weakened pathogen clearance.

Flores-Mehia et al. demonstrated that in SIRS, compared to healthy volunteers, higher levels of both pro-inflammatory (TNF-α, IL-1β, IL-6, and IL-8) and anti-inflammatory cytokines (IL-1Ra and IL-10) do not affect bacteria uptake performed by neutrophils, however phagosome maturation is decreased [121].

Tofacitinib may impair immune response to *Candida albicans*, among others, by impairing phagocytic capacity of neutrophils [122]. Drugs, which modulate neutrophils phagocytosis without targeting cytokine signaling, were reviewed elsewhere [123].

## 5. The Impact of Cytokines on NET Formation

The neutrophil activation, as well as NET formation, is cytokine-dependent with the most important cytokines described below (Table 3).

IL-1β is one of the “early” proinflammatory cytokines, also associated with increased mortality during sepsis. Time- and dose-dependent immunosuppressive agent—anakinra, shows positive correlation between blocking IL-1β receptor, NET formation, and IL-1β cytokine production. Suppressed expression of IL-1β receptor significantly reduced NET formation [124].

Yaqinuddin et al. showed that IL-1β/neutrophil extracellular traps feedback loop is present during SARS-CoV-2-induced acute lung injury. The authors noticed that both SARS-CoV-2 and sepsis are accompanied by IL-1β overproduction. They concluded overproduced IL-1β stimulates NET formation via activating NLRP3 inflammasome complex [125]. IL-1β produced by the NLRP3 inflammasome is a key inducer of NETs [131].

According to Tomar et al., SARS-CoV-2 infections may be accompanied by cytokine storm and sepsis [132], as mentioned above. Although the knowledge about triggers of the cytokine storm during SARS-CoV-2-induced sepsis is still not complete, authors hypothesized that the crucial role is played by neutrophils and their ability to form NETs [132]. They correlated appearance of IL-1β, and TNF-α with increased NET formation but the direct influence was not explored.

Multiple inducers of NETs have been reported; however, IL-8 seems to be the most effective stimulator of NET formation among investigated cytokines. Abrams et al. tested the influence of IL-8, IL-6, TNF-α, IL-1β, and selected histones on NET formation. A significant increase of NET formation was observed only for IL-8. When added to neutrophils, harvested from healthy volunteers, IL-8 induced NET formation. Conversely, incubation of healthy neutrophils with plasma obtained from septic patients attenuated NET formation by a functional anti–IL-8 blocking. Authors showed that IL-8-induced NET formation is dependent on Ras/Raf/MAPK pathways as ERK inhibition attenuates the effect and anti-IL-8 mAb diminishes ERK phosphorylation [126]. This study confirmed the role of IL-8 and MAP kinases in NET formation, but whether there are other molecules/pathways affected by IL-8 remains of interest.

A similar question was asked by Alsabani et al. [127]. Authors tested the influence of plasma obtained from septic patients and septic mice on NET formation by neutrophils isolated from healthy donors or mice, respectively. The treatment of healthy cells by septic plasma resulted in an increase of NET formation in both experimental models. Inhibition of CXCR1/2 (receptors of IL-8) using reparixin in septic mice reduced NET formation, which points to CXCR1/2 signaling-induced NET formation dependence [127].

Huang et al. compared wild-type mice group with wild-type mice sepsis group and detected a significant increase in TNF-α and IL-6 concentration comparing in the control. Increased NET formation was detected in the lung tissues in the sepsis group, which was significantly higher than in the control group, but authors did not correlate both observations [133]. Thus, convergence of both mechanisms remains unexplained.

Contrary to above-mentioned findings, Kaufman et al. did not observe any correlation between cytokines and nucleosomes or HNE-DNA [134]. Although the authors tested only IL-6 and TNF-α, they did not exclude influence of other cytokines.

Chrysanthopoulou et al. observed in the murine model of ferric chloride-induced thrombosis that IL-29 activates NETosis via mTOR inhibition [128].

Chemokine PF4 (CXCL4) can play a role in regulating in vitro human NETosis [135], and its recombinant form may also directly stimulate neutrophils [135]. In vivo, MKEY (a peptide inhibitor of CXCL4/CCL5 heterodimer formation) reduces NETosis in a model of acute lung injury [129]. Gollomp et al. showed that PF4 increased NET-mediated bacterial uptake and improved outcome in murine models of sepsis [130]. Little data exist on the distinction between neutrophils’ dysregulation in SIRS and CARS. During the former, as well as in sepsis, the apoptosis of neutrophils is inhibited, thus leading to increased tissue damage by the release of ROS and elastase [136,137,138]. The half-life of these cells, usually not exceeding 6 h, is markedly prolonged under pro-inflammatory conditions. Delayed apoptosis may be attributed to activated anti-apoptotic factors and NF-κB and further suppression of caspases 3 and 9 [139]. Accumulation of activated neutrophils is also associated with increased NETosis [140]. Under acute inflammatory circumstances, ICAM-1+ neutrophils and low-density neutrophils produce increased amounts of NETs [141,142].

Tofacitinib decreases NET formation [143]. Drugs, which modulate NET formation without targeting cytokine signaling, were reviewed elsewhere [123].

## 6. Conclusions

**Figure 1:**
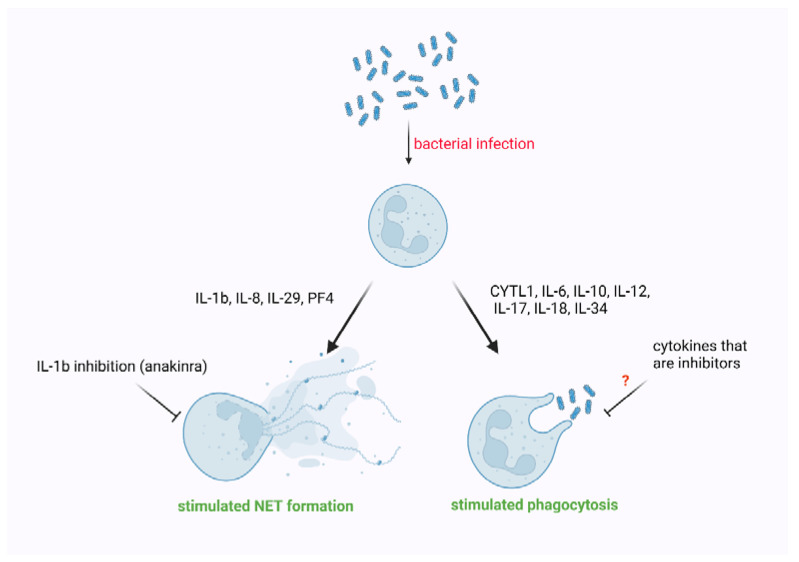
The effect of selected cytokines on phagocytosis and NET formation.

Even though sepsis is a life-threatening condition, the severity of which in a large part results from cytokine storm and collateral organ damage, our knowledge about roles of cytokines is still far from satisfying. Additionally, sepsis is not a single disease, but a condition resulting from an organism’s response to different microbes, which compromises the uniformity of in vivo studies. A significant limitation of in vitro studies is that usually single cytokines are tested, without considering significant interactions between different cytokines. Some drugs specifically target signaling mediated by particular cytokines. They were first introduced in rheumatology, but after the COVID-19 pandemic outbreak, their capacity to improve patients’ survival was validated. However, our understanding of the influence of cytokines’ signaling inhibition on neutrophil function is limited. Knowing the balance between “not enough” and “too much” regarding concentrations of crucial cytokines in sepsis progression may open new perspectives on novel therapies. Our review is the first to summarize the impact of tested cytokines on phagocytosis and NET formation during sepsis. We summarized our findings at Figure 1. Although many authors highlight cytokines responsible for stimulation of effector functions of neutrophils, further investigations are needed.

## 7. Study Selection

Although this is a narrative review, we ran a systematic search of the literature to ensure that most, if not all, necessary articles are identified. We searched for studies reporting outcomes of sepsis course, the importance of neutrophils during sepsis, and the impact of cytokines on phagocytosis and NET formation. All articles written in English, except for letters, reviews, and editorials, were included. We searched using electronic databases: PubMed, Web of Science, and Scopus. To find all relevant articles, we used a search engine specified in advance for each database, identifying 690 and 363 articles regarding phagocytosis and NET formation, respectively. Additionally, we screened references of selected articles to find papers not identified during the primary search. The collected publications were first screened by title and—if necessary—abstract by BG, then the full texts of previously accepted studies were assessed. In total, 15 publications for phagocytosis and NET formation were included and discussed in this review.

Search engines:

PubMed: (Neutrophils[mh] OR “neutrophil*” OR “polymorphonuclear leukocyte*” OR “polymorphonuclear cell*”) AND (cytokines[mh] OR “cytokine*”) AND (phagocytosis[mh] OR “phagocyto*”) AND (sepsis[mh] OR “sepsis” OR “septic shock”).

(Neutrophils[mh] OR “neutrophil*” OR “polymorphonuclear leukocyte*” OR “polymorphonuclear cell*”) AND (cytokines[mh] OR “cytokine*”) AND (extracellular traps[mh] OR “extracellular trap*” OR “NET” OR “NETs” OR “netosis”) AND (sepsis[mh] OR “sepsis” OR “septic shock”).

Web of Science: ((“Neutrophil *” OR “polymorphonuclear leukocyte*” OR “polymorphonuclear cell*”) AND “cytokine*” AND “phagocyto*” AND (“sepsis” OR “septic shock”)).

(((“Neutrophil*” OR “polymorphonuclear leukocyte*” OR “polymorphonuclear cell*”)) AND “cytokine*” AND (“extracellular trap*” OR “NET” OR “NETs” OR “netosis”) AND (“sepsis” OR “septic shock”)).

Scopus: TITLE-ABS-KEY ((“Neutrophil*” OR “polymorphonuclear leukocyte*” OR “polymorphonuclear cell*”) AND “cytokine*” AND “phagocyto*” AND (“sepsis” OR “septic shock”)) AND (LIMIT-TO (DOCTYPE, “ar”) OR LIMIT-TO (DOCTYPE, “re”)) AND (LIMIT-TO (LANGUAGE, “English”)).

TITLE-ABS-KEY ((“Neutrophil*” OR “polymorphonuclear leukocyte*” OR “polymorphonuclear cell*”) AND “cytokine*” AND (“extracellular trap*” OR “NET” OR “NETs” OR “netosis”) AND (“sepsis*” OR “septic shock”)) AND (LIMIT-TO (DOCTYPE, “ar”) OR LIMIT-TO (DOCTYPE, “re”)) AND (LIMIT-TO (LANGUAGE, “English”)).

## Figures and Tables

**Table 1:** The most important sepsis mediators.

Type of Mediators	Examples	Ref.
Pro-inflammatory cytokines	IL-1β, IL-6, IL-8, TNF-α, MCP-1, IL-1RA, TNF-R1/2, HMGB-1	[69,70,71]
Mediators of neutrophil activation	Proteins in neutrophil granules, CD64, CD11b, sCD14, TREM-1, HBP, sRAGE, TLR, suPAR, mHLA-DR	[72,73,74,75,76,77]
Acute phase proteins	CRP, procalcitonin, LBP, PTX3	[78]
Complement system	C3b, C5a	[79]
Mediators of immunosuppression in sepsis	mHLA-DR, CTLA-4, PD-1, IL-10, IL-1Ra	[77,80,81,82,83]
Mediators of organ injury	Lactate, NGAL, TIMP2, troponins, microRNAs (miR-181, miR-150)	[84,85,86,87,88,89]
Mediators of endothelium injury	ICAM-1, VCAM-1, E-selectin, VEGF	[90,91]
Coagulation activation	Antithrombin, thrombomodulin, C-protein, S-protein, D-dimers	[92,93,94]
Mediators of mtDNA injury	N-formylmethionine containing mitochondrial proteins (CYOX-S-1, CYOX-S-2), proteins not containing N-formylrnethionine (CYOX-S-IV, CYOX-S-V, CYOX-S-VI, Complex II), formyl-peptide receptor (FPR) and its variant FPRL1 (FPR-like 1), unmethylated CpG, P2X7 receptor	[95,96,97,98]
Other	MMP-9, lactoferrin	[99,100]

Abbreviations are explained in Appendix A.

**Table 2:** Cytokines secreted during sepsis that may be responsible for neutrophils’ phagocytosis.

Cytokine	Organism	Setting	Target	Effect	Ref.
CYTL1	in vitro Human, in vivo Mice	*Escherichia coli* infection	phosphorylation of protein kinase B (Akt)	enhanced phagocytosis	[113]
IL-6	Mice	*Staphylococcus aureus* infection	phosphorylation of STAT3	augmented the uptake of bacteria and phagosome acidification	[114]
IL-6	Human	*Staphylococcus aureus* infection	n.i.	enhanced phagocytosis and stimulated ROS generation	[115]
IL-10	Mice	*Escherichia coli* induced meningitis	increased expression of CR3	enhanced phagocytosis	[116]
IL-12	Mice	polymicrobial sepsis induced by cecal ligation and puncture (CLP)	induction of IFNγ	enhanced phagocytosis	[117]
IL-17	Mice	*Candida albicans* induced sepsis, and zymosan-induced multiple organ failure	n.i.	enhanced phagocytosis	[118]
IL-18	Mice	polymicrobial sepsis induced by cecal ligation and puncture (CLP)	n.i.	did not affect phagocytosis	[117]
IL-34	in vitro Human, in vivo Mice	sepsis	n.i.	enhanced phagocytosis	[119]

n.i.—not identified.

**Table 3:** Cytokines secreted during sepsis that may be responsible for neutrophils’ NET formation.

Cytokine	Organism	Setting	Target	Effect	Ref.
IL-1β	Human	(LPS)-induced and phorbol-12-myristate 13-acetate (PMA)-induced formation	n.i.	Increased NET formation	[124]
IL-1β	Human	SARS-CoV-2-induced acute lung injury, sepsis	NLRP3	Increased NET formation	[125]
IL-8	Human	Intensive care units (ICU), sepsis	Ras/Raf/MAPK	Increased NET formation	[126]
IL-8	Human	E. coli-induced sepsis	CXCR1/2	Increased NET formation	[127]
IL-8	Mice	Experimental sepsis (caecal ligation and puncture or intraperitoneal injection of E. coli)	CXCR1/2	Increased NET formation	[127]
IL-8	Human	n.i.	n.i.	Increased NET formation	[25]
IL-29	Mice	ferric chloride-induced thrombosis	mTOR	Activation of NETosis	[128]
PF4	Human	acute lung injury (ALI)	n.i.	Increased NET formation	[129]
PF4	Mice	Sepsis	n.i.	Increased NET formation	[130]

n.i.—not identified.

## Data Availability

Not applicable.

## Appendix A

The explanation of the abbreviations used at Table 1. IL-1β—interleukin 1β, IL-6—interleukin 6, IL-8—interleukin 8, TNF-α—tumor necrosis alpha, MCP-1—Monocyte chemoattractant protein-1, IL-1RA—interleukin-1 receptor antagonist, TNF-R1/2—TNF receptor-1, HMGB-1—High mobility group box 1, CD64—Cluster of Differentiation 64, CD11b—Cluster of Differentiation 11b, sCD14—plasma levels of soluble CD14, TREM-1—Triggering receptor expressed on myeloid cells 1, HBP—Heparin-binding protein, sRAGE—soluble Receptor for AGEs, TLR—toll-like receptor, suPAR—soluble form of the uPAR, mHLA-DR—human leucocyte antigen DR, CRP—C Reactive Protein, LBP—Lipopolysaccharide-binding protein, PTX3—Pentraxin-3, C3b, C5a—complement components C3 and C5, CTLA-4—cytotoxic T-lymphocyte antigen-4, PD-1—programmed cell death-1, IL-1—interleukin 10, IL-1Ra—IL-1 receptor antagonist, NGAL—Neutrophil gelatinase-associated lipocalin, TIMP2—tissue inhibitor of metalloproteinase 2, ICAM-1—intercellular adhesion molecule 1, VCAM-1—Vascular cell adhesion protein 1, VEGF—vascular endothelial growth factor, CYOX—cytochrome oxidase, MMP-9—Matrix metallopeptidase 9.
